# The use of Amerindian charm plants in the Guianas

**DOI:** 10.1186/s13002-015-0048-9

**Published:** 2015-09-15

**Authors:** Tinde van Andel, Sofie Ruysschaert, Karin Boven, Lewis Daly

**Affiliations:** Department Biosystematics, Wageningen University, PO Box 9517, 2300 RA Leiden, The Netherlands; WWF Guianas, Henck Arronstraat 63 Suite E, Paramaribo, Suriname; Ministry of Foreign Affairs, Government of the Netherlands, Bezuidenhoutseweg 67, 2594 AC The Hague, The Netherlands; Institute of Social and Cultural Anthropology University of Oxford, Oxford, UK

## Abstract

**Background:**

Magical charm plants to ensure good luck in hunting, fishing, agriculture, love and warfare are known among many Amerindians groups in the Guianas. Documented by anthropologists as social and political markers and exchangeable commodities, these charms have received little attention by ethnobotanists, as they are surrounded by secrecy and are difficult to identify. We compared the use of charm species among indigenous groups in the Guianas to see whether similarity in charm species was related to geographical or cultural proximity. We hypothesized that cultivated plants were more widely shared than wild ones and that charms with underground bulbs were more widely used than those without such organs, as vegetatively propagated plants would facilitate transfer of charm knowledge.

**Methods:**

We compiled a list of charm plants from recent fieldwork and supplemented these with information from herbarium collections, historic and recent literature among 11 ethnic groups in the Guianas. To assess similarity in plant use among these groups, we performed a Detrended Component Analysis (DCA) on species level. To see whether cultivated plants or vegetatively propagated species were more widely shared among ethnic groups than wild species or plants without rhizomes, tubers or stem-rooting capacity, we used an independent sample *t*-test.

**Results:**

We recorded 366 charms, representing 145 species. The majority were hunting charms, wild plants, propagated via underground bulbs and grown in villages. Our data suggest that similarity in charm species is associated with geographical proximity and not cultural relatedness. The most widely shared species, used by all Amerindian groups, is *Caladium bicolor*. The tubers of this plant facilitate easy transport and its natural variability allows for associations with a diversity of game animals. Human selection on shape, size and color of plants through clonal reproduction has ensured the continuity of morphological traits and their correlation with animal features.

**Conclusions:**

Charm plants serve as vehicles for traditional knowledge on animal behavior, tribal warfare and other aspects of oral history and should therefore deserve more scientific and societal attention, especially because there are indications that traditional knowledge on charms is disappearing.

**Electronic supplementary material:**

The online version of this article (doi:10.1186/s13002-015-0048-9) contains supplementary material, which is available to authorized users.

## Background

**Fig. 1:**
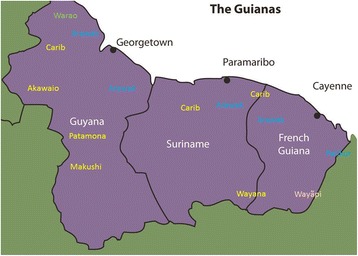
Map of the Guianas with schematic locations of the indigenous groups relevant to our study, based on the linguistic map of South America (http://www.muturzikin.com/cartesamerique/7.htm). Carib-speaking groups are indicated in yellow, Arawak-speaking groups in blue, Tupi-speaking Wayãpi in pink and the Warao-speaking group in green

Magical charm plants used to ensure good luck in hunting, fishing, agriculture, love and warfare are known among many different groups of Amerindians in the Guianas (Guyana, Suriname and French Guiana, see Fig. 1). These charms are grouped under the local terms *bina* (Arawak), *turara* or *moran* (Carib), *hemït* (Wayana), *muran* (Makushi), *murang* (Akawaio), *aibihi* (Warao), *polã* (Wayãpi), *masas* (Palikur) and *taya* by several people of indigenous and mixed origin [1–8]. Although they can consist of animal parts, items of material culture, symbolic tattoos, stones, and petroglyphs, the majority of these charms are plants [4].

As early as 1665, the Reverend Raymond Breton [9] documented the terms *tula:la* and *táya* in his dictionary of the Carib language spoken on the French Caribbean islands. He recognized them as plants of the Araceae family, “some of them having reddish leaves” … “used by all Indians, for magic purposes, especially to protect them against the Whites”. Not only were these *taya* plants used to heal the wounds caused by poisoned arrows, their juice was also mixed with the red paint made from *Bixa orellana* fruits and rubbed on the body to pacify the enemy [9]. A few years afterwards, the French plantation manager Jean Goupy des Marets mentioned the use of *toural* among the Indians of French Guiana in his diary [10]. The use of charms among Indians in Guyana and Suriname was first described by 19th and early 20th century ethnographers and missionaries [6, 7, 11–14]. These vegetable charms were thought to have substantial magical power, and were usually described as “fleshy arum-like plants with sagittate leaves used as a good-luck charm” [15] or “plants....that effect their purpose by enticing or attracting the particular object of desire yearned for, whatever it may be- from the capture of an animal to the gratification of a wish” [7]. In his research among Pemon Indians in Venezuela, Thomas [16] described *muran* as plants that were rubbed into cuts in the arms or legs to ensure success in hunting, which had potentially beneficial effects, but using them without proper instruction was potentially fatal.

According to Penard and Penard [6], charm plants always have an underground bulb, which is used alone or mixed with various animal organs (e.g., blood, brains, hairs or feathers) and rubbed on the hunter’s skin, his bow, arrow, fishing rod or dog, or simply carried in his pocket to have a greater chance to catch this particular animal. Every game animal or edible fish is said to have its own charm, of which the root or leaves resemble the color or shape of the desired animals fur, head or other organs [3, 7]. Most studies report that Amerindians procure their charm plants from the forest and transplant them to their house or garden [4, 7–17]. The charms are usually grown in pots or between the vegetation in a secret place to hide them from others and prevent menstruating women from touching or urinating over them, acts which would spoil their magic power [8, 18]. In their description of Surinamese Caribs, Penard and Penard [6] mention that “their cultivated ornamentals are nothing else than charms or *toelala*”.

Although extensively documented in the past and discussed by modern anthropologists in their role as social and political markers and exchangeable commodities [18], these symbols of both tradition and modernity [4] have hardly received attention by botanists. While the earliest herbarium vouchers from the Guianas were collected to document useful plants [19, 20], 20th century botany moved away from its roots in botanical gardens and applied botany and focused on the detection, description and classification of taxa [21]. Floristic studies in the Guianas have largely concentrated on wild plants [22, 23]. As a result, domesticated (crop) plants of the Guianas are underrepresented in herbarium collections [24], while information on plant cultivation and uses are often lacking from herbarium labels or, if documented by the collector, not entered in the databases of digitized vouchers hosted by herbaria.

In his description of Wayana charms, Chapuis [18] remarks that “it is not the plants themselves that motivate our work, but the discourses, practices and representations that are attached to them”, which may reflect a general attitude among anthropologists that voucher collection is not necessary [25, 26]. In ethnobotany, however, herbarium specimens vouch for the identity of the plants being studied, and are themselves documents of plant use by people in a given moment of time [21, 26]. The secrecy surrounding vegetable charms [4, 8], and the fact that aroids are difficult to process into herbarium vouchers [26], may also contribute to the underrepresentation of these plants in herbaria. The Naturalis herbarium (L) houses the world’s largest plant collection from the Guianas. The oldest collection of the Amerindian charm plant *Caladium bicolor* was made by F.L Splitgerber in 1837 and described as “*taayer. In Surinami cultis vulgaris*” (taya. Commonly grown in Suriname). Recent specimens include a few *taya* collected by Grenand et al. [5] in French Guiana and charm plants collected by Reinders [17] and Van Andel [8] in northwest Guyana.

It is likely that many of the vegetable charms that were once used by Amerindians in the Guianas have lost their function today. In their detailed description of Surinam Carib *toelala* (unfortunately without botanical collections), Penard and Penard deliberately chose not to describe the charm to prevent offspring: “incredible as it may sound, even civilized people often use this charm in Suriname” [6]. As there is no documentation of the use of this charm by modern Surinamese Caribs, the identity of this particular plant can no longer be traced. For some ethnographers, it seemed just too much work to document all the charms, as Gillin [27] notes: “A large number of plants are used as *binas*. I have made no attempt to exhaust the list of plants used for this purposes”. Others acknowledge that their list is not complete and call for further studies, such as Rodway [13]: “We may safely presume that we do not know a quarter of them [the charms]; yet I have thought it well to make a provisional list. The subject is so curious and interesting that possibly some people may be induced to go farther towards completing the collection”. When Chapuis [18] started his study, the Wayana told him that they only used hunting charms and had abandoned the use of warfare *hemït*. They lived in peace now, and growing such dangerous plants would only bring trouble.

The use of magic plants is not always easily combined with Christianity. Around 1906, an Arawak informant of Penard and Penard said that he did not know any *bina*, because he was baptized. When the ethnologists asked him how he could hunt without charms, he answered: “When we need hunting charms, we buy them from bad Caribs” [6]. A century later, when the Amazon Conservation Team offered to help the Wayana to set up a traditional health clinic in Palemeu, the village captain only agreed when the services were limited to medicinal plants for physical diseases; he wanted no *piyai* practices (shamanistic healing) in his clinic [28]. According to Grenand et al. [5], Palikur Indians have a rich medicinal flora, but use fewer charms than their neighboring Wayãpi, because of a long-term influence of diverse Christian churches, who see these plants not as herbal remedies but more as poisons or black magic. The evangelical Church of Christ and Christian Brethren denominations prohibit the use of charms among members of their congregation in Makushi villages, which results in people rejecting their use and denying their efficacy, at least publicly [4]. In northwest Guyana, practicing shamans and their ritual knowledge have almost completely vanished, while youngsters are reluctant to learn the tools of the trade. In most communities, the information on plant charms is scattered among elders, who are often not aware of each other’s knowledge [8]. Given the scant ethnobotanical documentation and the ongoing loss of knowledge regarding plant charms in the Guianas, documenting their present use is of great importance.

The aim of this paper is to make a comparison in the use of charm species among different indigenous groups in the Guianas, based on recent fieldwork in Suriname and Guyana, information on herbarium labels, recent and historic literature (Table 1). We formulated the following research questions:

**Table 1:** Data sources

Ethnic group	Country	Data sources	Main references
Akawaio	Guyana	literature	[3, 36]
Arawak	Guyana	fieldwork Van Andel, herbarium vouchers, literature	[17]
Arawak	Suriname	fieldwork Ruysschaert, herbarium vouchers, literature	[6]
Carib	Guyana	fieldwork Van Andel, herbarium vouchers	
Carib	Suriname	literature	[6, 11]
Macushi	Guyana	fieldwork Daly, photographs	
Palikur	French Guiana	literature, herbarium vouchers	[5]
Patamona	Guyana	literature	[37, 66]
Warao	Guyana	literature, herbarium vouchers	[17]
Wayana	French Guiana, Suriname	fieldwork Boven, photographs, literature	[18]
Wayãpi	French Guiana	literature, herbarium vouchers	[5]

1. Which species are most widely used by Amerindians in the three Guianas and why?
2. Are these plants collected from the wild or cultivated?
3. Is similarity in charm species related to geographical or cultural proximity?

We hypothesize that cultivated plants are more widely shared than wild plants, as the natural vegetation of the Guianas is not homogeneous. We also expect that charms with tubers, rhizomes or stems that can be easily propagated by cuttings are more widely used than those without such underground organs or stem-rooting capacity.

## Methods

We compiled a list of plant charms from ethnographic, anthropological and ethnobotanical literature on 11 indigenous groups in the Guianas, counting the Guyanese Arawaks and Caribs separately from the Surinamese Arawaks and Caribs. We compiled published and unpublished fieldwork data, collected in Suriname among Arawaks by Ruysschaert in 2004–2006 and among Wayana by Boven in the early 1990s, and in Guyana among Makushi by Daly in 2011–2013 and among Caribs by van Andel in the late 1990s. We supplemented these data with recent and historic literature on charm use and information on (digital) herbarium labels.

During fieldwork, we followed the Code of Ethics of the International Society of Ethnobiology [29]. All participants were informed of our intent prior to any interview and their verbal or written permission was obtained. Voucher collection was done after obtaining the necessary collection permits from the governments of Guyana and/or Suriname. Vouchers were deposited at the herbaria in Paramaribo (BBS), Georgetown (BRG), Ghent (GENT) and Naturalis (L). We identified plant specimens from photographs, literature descriptions and physical herbarium vouchers at the Naturalis herbarium, as well as digital images of collections at Naturalis [30], the Missouri Botanical Garden Herbarium [31], and l‘Herbier IRD de Guyane in Cayenne [32]. A few vouchers of plant charms collected in the 1990s and stored in the Naturalis herbarium were given new identifications. Domestication status of species and specimens was retrieved from the botanical literature, field observations, herbarium labels and the Checklist of the Guiana Shield [33]. Current scientific and author names were checked by means of the Plant List [34].

We listed all reports on plant charms in an Excel table (Additional file 1), with vernacular names, scientific names and family (when this could be established with certainty), short cultivar description, associated animals, collection numbers (when available) and data sources, and classified the charms in eleven categories, largely based on those defined by Daly [4]: charms for hunting, fishing, agriculture, working, learning, love, luck, protection against (supernatural) enemies, and charms to do evil, in the literature often referred to as *kanaima* charms [4, 35, 36]. We used the original spelling of vernacular names from the publications, although this often did not follow the official spelling of these indigenous languages.

To assess the similarity in plant use between countries and groups, and see whether this was associated with geographical or cultural proximity, we performed a Detrended Component Analysis (DCA) on species level. We defined geographical proximity here as groups living close to each other (neighboring tribes), which facilitates the exchange of ethnobotanical knowledge. Cultural proximity was defined as groups belonging to the same language group (and thus sharing a cultural origin), but not necessarily living at a close distance of each other. To perform our DCA, charms that could be linked with a reliable degree of certainty by species or genera were listed as separate species in a presence-absence data matrix with species in rows and 11 indigenous groups in columns. Unidentified plants were excluded and all landraces or cultivars within a species were counted as one species. All plant species used as charms by an indigenous group were used as the sample unit in our analysis. We plotted the results of our DCA analysis on the two main axes that caused the distribution of the data to visualize potential overlap and variation in plant use by the 11 groups. All analyses were performed in PC-ORD 5.0.

To assess the most widely used charm species, their domestication status and the presence of plant organs that facilitated vegetative reproduction, we constructed another matrix in which we listed for each species whether it was wild or cultivated. Within cultivated plants, we distinguished domesticated plants (plants that do not occur in the wild but need to be grown by humans, such as crops) and plants that are taken from the wild and grown around houses or in forest gardens [37]. In the same matrix, we listed whether the species possessed organs that facilitated vegetative propagation. To see whether cultivated plants or vegetatively propagated species were more widely shared among ethnic groups than wild species or plants without rhizomes, tubers or stem rooting capacity, we compared the mean number of ethnic groups using the two groups of species (wild vs. cultivated and easily propagated species vs. not so) by using an independent sample *t*-test. All statistical tests were done with the program IBM SPSS 19.0.

## Results

### Diversity in plant charms

“There are charms for making people energetic workers, for making women's hair grow long; making people plump; enabling the shaman to bring down certain spirits during his séance; making someone love you; making children grow tall; enabling you to sing well, and to achieve many other things”. This description of Butt [3] on the use of charms among the Akawaio illustrates the diversity of magic plant use among indigenous groups in the Guianas. Unfortunately, the fact that she only lists six charm plants, of which only three could be identified to species level with some degree of certainty, is also illustrative for the scanty botanical documentation of Amerindian charms by anthropologists. Still, we retrieved a total of 366 charm types from literature and unpublished fieldwork data from the Guianas. In the Additional file 1, we list all charm records with literature references, herbarium collection numbers, vernacular names and types of charms. Although we were unable to identify many charms from the older literature, we included them in list to facilitate future research.

Most of the documented charm types were hunting charms (127 records), followed by protection charms against bad spirits or other (super-) natural enemies (83 records), love charms (50), charms for fishing (34), luck (26), and protection against snakes (21). A striking feature is the large number of *Caladium bicolor* cultivars that vary in color, variegation pattern, leaf and tuber shape and size (Additional file 1). *Maranta arundinacea* has only two cultivars (purple and green), but *Xanthosma sagittofolium* also appears to be represented by several cultivars. For the lilies within the genus *Hippeastrum* it was not possible to ascertain whether the various charms represented different species or several phenotypes within a species, as few of the photographs and botanical collections had flowers.

### Most widely used charm species

The 366 charm records represented at least 145 plant species, of which 10 were used by three or more ethnic groups (Table 2). The most widely used species was *Caladium bicolor*, used as a charm by all 11 indigenous groups, followed by *C. schomburgkii*, *Maranta arundinacea* and *Eleutherine bulbosa*. The largest number of charm species was reported among the Suriname Arawaks, followed by the Makushi in Guyana. It should be noted that Table 2 lists the charms per species only; the highest number of charm types was reported among the Surinamese Caribs in the early 1900s [6, 11], but many of these plants could not be identified and they may include several cultivars of *C. bicolor* (Additional file 1). The remark of Grenand et al. [5] that the Palikur used much fewer charms than their neighboring Wayãpi because of their conversion to Christianity is contradicted by the number of charm species that were reported by the same authors: 19 Palikur charms vs. 4 Wayãpi species (Table 2). According to the Brazilian anthropologist Joana Cabral de Oliveira (personal communication February 2015), the Wayãpi of the Amapa state in Brazil still use a wide range of plant charms, mostly Araceae and particularly hunting charms.

**Table 2:** Most frequently used charm species among the 13 ethnic groups in the Guianas, their domestication status and possibilities for vegetative propagation

Species	Domestication	Vegetative	Akawaio	Arawaks	Arawaks	Warao	Carib	Carib	Macushi	Wayana	Patamona	Palikur	Wayãpi	Total
	Status	Propagation	Guyana	Guyana	Suriname	Guyana	Guyana	Suriname	Guyana	Suriname	Guyana	French Guiana	French Guiana	
*Caladium bicolor*	C (W)	1	1	1	1	1	1	1	1	1	1	1	1	11
*Maranta arundinacea*	D	1			1	1	1	1	1	1	1			7
*Eleutherine bulbosa*	C (W)	1		1	1	1	1		1	1				6
*Zingiber zerumbet*	C	1		1		1		1			1			4
*Caladium schomburgkii*	C (W)	1			1	1	1	1					1	5
*Capsicum annuum*	D	0			1		1	1						3
*Cyperus articulatus*	C (W)	1		1			1		1					3
*Hippeastrum puniceum*	C (W)	1	1		1			1	1			1		5
*Xanthosma brasiliense*	C	1			1		1	1	1	1				5
*Abelmoschus moschatus*	D	0					1	1						2
*Caladium humboldtii*	C	1		1		1	1							3
*Lycopodiella cernua*	W	0			1						1			2
*Mimosa pudica*	C (W)	0			1							1		2
*Montrichardia arborescens*	W	0			1				1					2
*Protium heptaphyllum ssp. heptaphyllum*	W	0			1				1					2
*Scoparia dulcis*	C (W)	0			1							1		2
Total		50	6	17	59	13	14	20	34	8	16	19	4	

A number 1 signifies documented use.

*D* Domesticated;*W* Wild; *C* Cultivated (not occurring in the wild in the Guianas); *C (W)* Taken from the wild, but cultivated in village or agricultural field

### Similarity in plant use across the Guianas

**Fig. 2:**
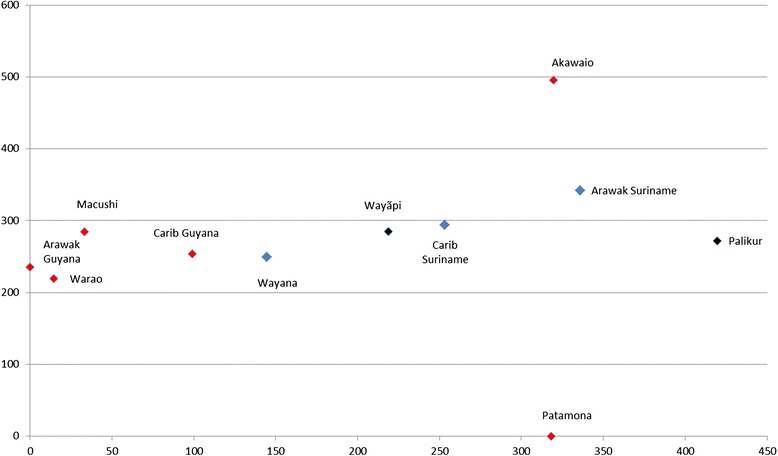
DCA scatterplot showing similarity in charm use on species level. Data points represent all charm species used per ethnic group. Clustered datapoints indicate similarity in plant species used. Blue datapoints represent Suriname, red ones Guyana and black ones French Guiana. Axes do not represent variables but serve to visualize variation and similarity in plant use

When results of our DCA analysis are plotted in a two-dimensional scatter plot (Fig. 2), we see that some ethnic groups resemble each other more than others with regard to charm species used (not taking into account the various cultivars). The closer two points are to each other, the more species they have in common. Generally, Fig. 2 shows that similarity in plant use is associated with geographical proximity. Except for the Patamona and the Akawaio, the Guyanese groups cluster closely together. The Patamona are a clear outlier, as they use a quite distinct set of charm species (Additional file 1). The remote position of the Akawaio, however, may be caused by a lack of ethnobotanical research rather than by differences in plant uses. The Surinamese groups also cluster together, but less tightly than most Guyanese groups. The Wayãpi are quite separated from the Palikur, although they both reside in French Guiana.

Figure 2 also indicates that a shared cultural origin does not necessary imply a similarity in plant use. Caribs and Arawaks, bordering groups in Guyana and Suriname, show more similarity to each other than to their cultural counterparts in the neighboring country. The Carib-speaking groups (True Caribs, Makushi, Akawaio, Patamona, and Wayana, see Fig. 1) do not cluster together and neither do the Arawak-speaking groups (true Arawak and Palikur). The Tupi-speaking Wayãpi do not stand out, and neither do the Warao, who speak an isolated language. Warao Indians were quoted to have learned all charms from Caribs or other indigenous groups [7, 17] which seems to be confirmed by our DCA analysis, as their plant use is quite similar to their close neighbors in Guyana (Arawaks and Caribs). In the case of the Patamona, geographical proximity is not associated with similarity in plant use. According to the Makushi interviewed by Daly [4], the neighboring Patamona were their enemies, which might explain the limited number of shared species (thus a lack of exchange in ethnobotanical knowledge).

### Cultivation of charms

Of the 145 charm species documented for the Guianas, 63 are truly wild, while 51 species are taken from the wild and grown in house yards or agricultural fields for magic (and sometimes other) uses. Another 31 species do not occur naturally in the Guianas according to the regional checklist [34]. Several of them are domesticated, agricultural crops (like *Zea mays* and *Maranta arundinacea*), others were probably taken from the wild in Venezuela or Brazil and brought by Amerindians to neighboring tribes in the Guianas to be grown as charms. Examples are the *Aristolochia* vine that was encountered in home gardens in northwest Guyana, but did not bear any resemblance to the *Aristolochia* species observed in the wild in the Guianas as described by Feuillet and Poncy [38]. However, the leaf surface and vein structure of the voucher specimen (TVA1008) strongly resembled a photograph of the *kami bina* taken by Daly in a Makushi village. Both are very similar to the species *Aristolochia odoratissima*, widely distributed from the Caribbean to Venezuela and Paraguay, but not occurring in the wild in the Guianas. In the 1840s, however, *A. odoratissima* was cultivated in Guyana [39] and grown in a garden in French Guiana in the late 1700s [38, 40]. The present individuals in Guyana prove that the species is still sporadically cultivated in Guyana.

Several indigenous informants confirmed that charms were brought by visiting Amerindians from elsewhere. Arawaks in Moruca mentioned that their cultivated individual of *Hymenocallis* cf. *littoralis* was brought in 1951 ‘from outside by Spanish Arawaks’ (probably from Venezuela). In Suriname, Powakka Arawaks said their *leguaan taja* (*Colocasia* sp.) was brought from the Carib village of Cabendadorp, located some 30 km away. A cultivar of *Xanthosoma sagittifolium* used as hunting charm was taken along by Wayãpi from Amapari (Brazil) when they visited their family in French Guiana [5]. Among the Makushi, charm plants are a key currency in exchange networks that span large distances; some of the plants in Guyana were brought from Makushi villages in Brazil. The Patamona charm *kobita*, used by avenging *kanaima* shamans to torture their victims, was identified as *Philodendron canaimae* [37]. This wild plant only has known occurrences in Venezuela [41], but the species name refers to the area where it was found, Canaima National Park in the Venezuelan Bolivar province [42], rather than to its use by the malevolent *kanaima* of the Patamona. Since the photograph of the *kobita* charm supplied by Whitehead [37] depicts a form of *Caladium bicolor*, the use of *P. canaimae* among the Patamona is questionable.

### Vegetative propagation

Of the 145 charm species documented for the Guianas, 50 have a rhizome or a tuber that facilitates long distance transport and vegetative propagation. Another 11 species (e.g., *Portulaca* spp., several Commelinaceae) do not have such a tuber, but can easily be grown from stem cuttings, as they quickly root at the nodes. Charm plants that can be reproduced by cuttings or tubers were used by a higher number of ethnic groups than plants that lacked such traits (Table 3). The many different forms of *Caladium bicolor* can be cloned from their tubers. This ensures that the offspring has a similar shape, color and variegation as its parent, and thus can serve as a charm to attract the same animal. Sowing *Caladium* seeds would probably yield a large variety of leaf shapes and colors [43], which would cause confusion on their specific uses. The cultivation of charms also enables their users to increase their influence over them. Several studies reported that Amerindians ‘fed’ their charms with blood of game animals or cassava beer, or made them small offerings like coins and cigarettes. This would either ‘train’ them or ‘make them happy’ [6], both of which increases their efficacy. Some plants are said to be so powerful that they can kill onlookers merely from the sight [4].

**Table 3:** Prevalence of charm types among the 11 ethnic groups, expressed as the mean number of groups using a particular species

Vegetative propagation	Number of species	Number of ethnic groups [mean ± standard dev.]
No	84	1.94 ± 1.01
Yes	61	2.43 ± 1.99*
Domestication status		
Cultivated and domesticated	82	2.50 ± 1.79
Wild	63	1.68 ± 0.88*

*Significantly different from the value on the previous row above (*p* < 0.05)

Cultivated species were also used by a higher number of ethnic groups than plants that were only collected from the wild. Not all cultivated species could be vegetatively propagated: some needed to be sown from seeds. Of the 82 cultivated species, only 32 were domesticated (agricultural) species, or wild species brought from outside the Guianas. The rest (50 species) were plants that occurred in the wild, but were actively taken home to be grown there, ensuring their availability when needed. Our results show that the domestication of wild plants for human use is still an ongoing process in the Guianas.

## Discussion

### Medicine or charm?

In Amerindian terminology, there is not always a sharp distinction between magic charms and herbal medicine [18]. Surinamese Arawaks squeeze the juice from *jeberu bina* (‘sore eye charm’, *Drosera capillaris*) into their eyes to cure conjunctivitis [44], while the leaves of *ebesere-bina* (‘foot fungus charm’, *Xiphidium caeruleum*) is applied as a remedy for athlete’s foot. In the vernacular names for these species, the term *bina* (charm) refers to a physical medicine instead of a magic charm. There is also a great deal of interchangeability among the Akawaio in the use of *murang* (charm) and *dibik* (medicine), although the latter corresponded more to the Western definition of ‘medicine’ [3]. The same was noted among the Makushi: some charms (*muran*) could also act as cures, while some medicinal plants (*epik*) acted as a charm. Another explanation for the vernacular names of medicinal plants used for physical illnesses that refer to charms may be that the healing properties of these plants are so effective that they received a magic connotation. Some indigenous groups clearly separate the two types of plants: the Wayãpi, for example, make a strict distinction between *poã* (herbal medicine) and *polã* (charms) [5].

### The mythical origin of plant charms

According to a widespread Carib and Arawak myth, people discovered the plant charms when they killed a large *Boa constrictor* because it had devoured several persons. The carcass of the snake, known by Caribs as *Orupéri* or *Aramári* [7] and by Arawaks as *Ololi* or *Kolekonáro* [45], was covered with leaves and carefully burnt. From its ashes sprouted different types of tuberous plants: mostly (but not all of them) aroids. Their bulbs were taken home and grown by the Indians to be employed as charms [7]. They found out which plant served to attract which animal by means of trial and error. A hunter would take a leaf or a tuber with him into the forest. If he would meet a type of game several times, then that animal would be attracted to the specific plant he carried along. When he encountered a jaguar or a venomous snake, he would throw away the charm and no longer cultivate it [7, 17]. Charms could also be trained by planting them in the burned remains of a game animal [6]. The Wayãpi in French Guiana believe that charms are not planted by people but by spirits, and that they grow from corpses of both animals and (evil) humans [5].

The Makushi in southern Guyana have a different story on the origin of charms: they were created by the twin heroes *Insikiron* and *Anike*, who were responsible for forging many elements of the world as it exists today [4]. According to the Wayana, living on the southeastern border of Suriname and French Guiana, charms (*hemït*) were first planted in ancestral times by their creator *Kujuli* on the inselbergs of the Tumuc Humac Mountains [18]. Later, these charms were intensively used (and planted) by the 18th century Wayana warrior Kailawa, who used them in his violent encounters with neighboring tribes [1].

### Biological explanation of charm characteristics

Common elements in these Amerindian myths are the presence of tubers, and the transport and cultivation of wild plants that naturally occur in burned, nutrient-rich locations. According to Penard and Penard [6], “only aroids can grow on the remains of a rotten animal”, although they later explain that “the entire soil is full of seeds, which are just waiting for the right moment to germinate, which mostly happens after burning or plowing the terrain”. According to Gillin [27], “a rank growth of leaf plants is often seen to cover the spot where a carcass has lain a few weeks before”.... “due to fertilization of the soil offered by the decay of a large amount of organic matter”. Tuberous herbs may indeed be the first plants that come up after burning a patch of forest, as their underground parts are insulated from lethal temperature and thus unharmed by the fire, sprout back again, stimulated by the enhanced light conditions and the increased availability of organic matter [46].

According to Ahlbrink [11], the most important reason why charms are mostly aroids, is that “*taja* grows easily, everywhere and occurs in countless varieties, different shapes and colors of leaves, spots, and the tubers also vary widely in shape. So in this family you will always find a variety that resembles the animal that you want to hunt or charms the person you desire”. Indeed, hundreds of commercial cultivars of *Caladium bicolor* have been developed by plant breeders [43, 47], although the types used in the Guianas should be considered as local landraces, bred from wild individuals by Amerindians and maintained over the centuries by vegetative cloning. Selection on shape, color and size of leaves and tubers and their consequent association with the diversity of Amazonian game animals an analogy of resemblance, also known as Doctrine of the Signatures, makes *C. bicolor* the ideal hunting charm. As discussed before, Amerindian myths concerning the origin of plant charms often refer to a trial and error process, in which the hunter learns which plant characteristics should be linked to which desired game species. This supports the theory that signatures are post hoc attributions rather than a priori clues to the utility of plants, and that Doctrine of the Signatures should be seen as a memory aid that serves to disseminate information [48].

Unfortunately, the variation in shape, size and color patterns of *C. bicolor*, and the lack of up-to-date reference material which allows for a correct identification of cultivars and landraces, makes them difficult to identify below species level, even when voucher material is available [43]. Further ethnobotanical inventories are needed on present-day charm use, backed up by herbarium vouchers, preferably combined with research on the morphological differences and genetic background of various landraces of *C. bicolor*. This could clarify the resemblances in species and landrace use among different ethnic groups and unravel the routes of exchange of plants and traditional knowledge regarding plants and animals among Amerindians in the Guianas.

### Loss in knowledge

Apart from rubbing a hunter’s body with the *Caladium bicolor* tuber, Surinamese Caribs in the early 1900s also collected the kidneys, heart or brains of the desired game animal, together with some organs of birds that either made similar noises or were otherwise associated with the desired animal [6]. These organs were then burnt with sand from the animal’s tracks and pounded into powder, which needed to be rubbed on the hunter’s body with the tuber of the specific charm. The hunter also had to perform complicated rituals linked to the game animal’s behavior, like rolling in the mud and making whistling sounds in case of a tapir. Descriptions of such elaborate rituals are lacking from recent anthropological and ethnobotanical studies in the Guianas. The modern hunter just rubs the tuber on his limbs or gun or carries it in his pocket, so it seems likely that these extensive ceremonial practices have died out. Ritual plant use is considered as a form of adaptive management of natural resources and serves as a vehicle for the transmission of traditional ecological knowledge [49]. An in-depth knowledge of a game animal’s morphology, behavior, food preference and its ecological association with other forest animals obviously forms the basis for a successful Amerindian hunter, rather than the aroid bulbs he cultivates. The latter, however, serve as a way of safeguarding and transferring his specialist knowledge.

During formal education in Guyana, no attention is paid to local Amerindian knowledge. Although traditional knowledge is being lost, modern charms recently appeared among the Makushi: a cash *bina*, shopping *bina*, blackman *bina*, Georgetown *bina*, Brazil *bina*, and a gold mine *bina* [4]. As the political, economic, and ecological concerns of contemporary life have changed for the Makushi, so too have the target objects, dispositions, and capacities of their charms [4]. The fact that charm mixtures are sold within Amerindian communities and to outsiders [8, 18] may contribute to the preservation of traditional knowledge as well.

Hunting charms are commonly planted in house yards, and knowledge regarding their cultivation and use is shared within families, friends, and neighbors, but charms that are used for cursing or other malevolent ends are more secretly guarded [4, 18]. The Wayana used to have several warfare charms that were planted in the forest and not sold or exchanged with others [2, 18]. Now the Wayana live in peace with their surrounding tribes, they deliberately chose to forget these dangerous plants, so the criminal *hemït* are no longer in use. According to Chapuis [18], these plants were probably poisonous and could either kill an enemy or make him gravely ill. Later he argues that these “charms that could turn men into killing machines were so secret that they may not have existed at all’, and formed “perhaps a botanically empty class, devoid of content material, but brimming with an overflow of social meaning” [18].

The Swedish biologist Daniel Rolander [50], however, described these Amerindian killing charms in his Surinamese diary (1754–1756). The extremely bitter sap of *Tabernaemontana citrifolia* was drunk to drive away sleeplessness and increase the courage of soldiers. “When taken in a generous dose, [the Amerindians] become almost berserk and go to meet the enemy with incredible bravery”, he wrote on 2 August 1755. The white latex of this forest tree causes dermatitis and systemic toxicity [51], indicating its potentially lethal properties. Barama River Caribs reported in 1996 that ‘bad people’ sometimes killed their enemies with *Malouetia flavescens* [8], another toxic genus within the Apocynaceae [52].

Interestingly, several of the tuber-producing species reported as charm plants throughout the Guianas were once grown as food crops. Domesticated in pre-Colombian times for their edible starch [53], *Calathea allouia*, *Maranta arundinacea* and *Canna indica* largely lost their function as food, as did the cultivars of *Xanthosoma sagittifolia* used as charms among the Wayana [2] and Wayãpi [5]. They belong to the same species as the domesticated *tannia* (Guyana), *tayerblad* or *pom tayer* (Suriname) or *chou Caraïbe* (French Guiana), widely grown for its edible leaves and starchy corm. Why these ancestral food crops transformed into ritual plants remains unknown, but this shift in use has certainly contributed to their survival.

The spiny, edible tubers of *Calathea ovata*, known in the 1950s by the Wayana as *pisoi* and collected for food [54, 55] were probably the same species as the charm cultivated by Arawaks and Caribs to catch marails in the early 1900s [6, 11]. Their use as food was either unknown or forgotten by coastal Amerindians. Strikingly, *Calathea ovata* was reported in the 1980s as a cultivated, edible plant with the name *pisoy* among Marowijne Maroons, descendants of escaped African slaves [56].

It has been argued that in many cases, New World Africans became the custodians of Amerindian botanical knowledge in the Caribbean [57]. Maroons in Suriname use many Amerindian domesticates (e.g., *Bixa orellana*, *Maranta arundinacea*) in their rituals to honor Amerindian spirits [58], while their ritual use among neighboring Amerindians has been lost. Rolander [50, 59] repeatedly mentioned plants with ‘a bad reputation’ among Amerindians, without disclosing the reasons why. One of these species (*Heliconia psittacorum*) is now used in Maroon rituals to pacify an Amerindian spirit [58]. The Amerindian use of this species (apparently still existing in the 1750s) is no longer remembered today, but given its reputation, it may have been a war charm. Magic war medicine has played a large role in the establishment of Maroon communities in the 18th century [60, 61]. During the Surinamese civil war in the 1980s, Maroon rebels searched for ancient war charms in interior communities [62]. Charms to become invulnerable to hostile attacks are still popular among Maroons today [58]. It is likely that some of these lost Amerindian war charms can be found among today’s Maroons.

### How do these charms function?

Ritual plant use is often said to work only on the psychological level, while potential pharmacological effects are frequently overlooked [49]. Apart from acting as an aid to gain self confidence in hunting, pursuing luck or confronting one’s enemies [11, 58] the hunter may disguise his own scent by rubbing the juice of aroid tubers on his body [6]. Especially if he mixes the tuber with organs of the desired species that he has caught earlier, he may take over the scent of the game animal itself and thus attract the other members of the herd. The scent of the aroids themselves is also said to attract game [11]. This can be explained by the fact that some forest animals, like peccaries, feed almost equally on roots, tubers and seeds that they find by uprooting the forest soil [63]. Some hunting charms used to be employed as hunting poisons elsewhere [64], like the *Hippeastrum* bulbs that were brought by Brazilian Wayãpi to their French Guianese tribesmen [5].

Plants used for evil doing or decreasing the power of an enemy can simply act as poisons, such as the charms that are secretly mixed into cassava beer [5] and the slow-acting, often fatal *kanaima* charms of the Patamona [37]. Plants that are rubbed into a dog’s nose may act as a nasal and chest decongestant and improve its ability to follow a scent [65]. Many aroids, including several *Caladium*, *Xanthosoma*, *Philodendron* and *Dieffenbachia* species, contain the toxic calcium oxalate, which causes intensive burning and itching on the mouth, throat and skin [51]. Rubbing the juice from aroid tubers into skin incisions, required for the effectivity of many Amerindian charms, must be quite painful. Most of the Patamona charms were prepared by drinking a decoction of boiled barks or leaves, followed by intentional vomiting [66]. Such emetics are also taken by Makushi shaman’s apprentices. These practices may simply be rituals that hunters or shaman trainees have to endure to display their strength and to prepare for pain and danger during the hunt or ritual tasks [36]. However, as the use of hallucinogenic plants to improve hunting success is well documented in other parts of the Amazon [7, 67], potential pharmacological effects on charm use in the Guianas should not be overlooked. Evil charms that are used to curse enemies from a distance, without coming into physical contact with the victim, often represent plants that were used in the past to poison people, but now only retained their symbolic power [58]. The Makushi cursing bina (*Dieffenbachia seguine*) was used in the colonial era to punish slaves: after being forced to eat the leaves, they would choke because the plant’s calcium oxalate crystals caused a fatal swelling of their throat [44].

## Conclusions

Our inventory of plant charms throughout the Guianas revealed that at least 145 species of charms are used, predominantly hunting charms. The most widely used are plants of wild origin and cultivated by means of vegetative propagation via tubers or rhizomes. The most frequently used species is *Caladium bicolor*, whose tubers allow for easy transport and whose natural morphological variability permits associations with a diversity of game animals, following the Doctrine of Signatures. Human selection on shape, size and color of the different landraces within Araceae and Marantaceae through clonal reproduction has ensured the continuity of morphological traits and their association with the features of animals. Carrying a bulb from a specific charm landrace that can be grown into an identical plant as the one left back home, facilitates the transfer of specific knowledge much better than wild plants (that may not be available around the village that is visited) or species that need to be grown from seeds. Charms can therefore be seen as vehicles for the transmission of ecological knowledge on plants, animals, and their complex interactions, but also of historical and cultural knowledge, exceedingly valuable in traditional cultures. Our research results show that similarity in plant use is related to geographical rather than cultural proximity. As there are strong indications that traditional knowledge regarding plant charms is disappearing, *in-situ* conservation of charm species and landraces in botanical heritage gardens should be considered, combined with the documentation and local appreciation of associated indigenous knowledge. We hope that our overview on charm use contributes to the conservation of this valuable biological and cultural resource.

## Additional file

Additional file 1:
**All reports on plant charms in the Guianas, retrieved from fieldwork, historic and recent literature and their characteristics.** (ODS 48 kb)
